# Personality and complex brain networks: The role of openness to experience in default network efficiency

**DOI:** 10.1002/hbm.23065

**Published:** 2015-11-26

**Authors:** Roger E. Beaty, Scott Barry Kaufman, Mathias Benedek, Rex E. Jung, Yoed N. Kenett, Emanuel Jauk, Aljoscha C. Neubauer, Paul J. Silvia

**Affiliations:** ^1^ Department of Psychology University of North Carolina at Greensboro Greensboro North Carolina USA; ^2^ Department of Psychology University of Pennsylvania Philadelphia Pennsylvania USA; ^3^ Department of Psychology University of Graz Graz Austria; ^4^ Department of Neurosurgery University of New Mexico Albuquerque New Mexico USA; ^5^ Department of Cognitive Linguistic, and Psychological Sciences, Brown University Providence Rhode Island USA

**Keywords:** default mode network, individual differences, personality, structural equation modeling, network science

## Abstract

The brain's default network (DN) has been a topic of considerable empirical interest. In fMRI research, DN activity is associated with spontaneous and self‐generated cognition, such as mind‐wandering, episodic memory retrieval, future thinking, mental simulation, theory of mind reasoning, and creative cognition. Despite large literatures on developmental and disease‐related influences on the DN, surprisingly little is known about the factors that impact normal variation in DN functioning. Using structural equation modeling and graph theoretical analysis of resting‐state fMRI data, we provide evidence that Openness to Experience—a normally distributed personality trait reflecting a tendency to engage in imaginative, creative, and abstract cognitive processes—underlies efficiency of information processing within the DN. Across two studies, Openness predicted the global efficiency of a functional network comprised of DN nodes and corresponding edges. In Study 2, Openness remained a robust predictor—even after controlling for intelligence, age, gender, and other personality variables—explaining 18% of the variance in DN functioning. These findings point to a biological basis of Openness to Experience, and suggest that normally distributed personality traits affect the intrinsic architecture of large‐scale brain systems. *Hum Brain Mapp 37:773–779, 2016*. © **2015 Wiley Periodicals, Inc**.

## Introduction

Neuroimaging research has increasingly focused on examining large‐scale structural and functional brain networks [Sporns, 2014]. One of the most widely studied networks is the default network (DN), a set of midline and inferior parietal brain regions that show increased metabolic activity in the absence of external task demands [Buckner et al., 2008; Greicius et al., 2003]. Since the initial discovery of the DN, researchers have sought to clarify its role in attention and cognition. DN activity is presumed to reflect spontaneous and self‐generated cognitive processes, such as autobiographical memory retrieval, episodic future thinking, theory of mind reasoning, mental scene construction, moral decision making, creative cognition, daydreaming, and mind‐wandering [Andrews‐Hanna et al., 2014; Beaty et al, in press; Fox et al., 2015; Stawarczyk and D'Argembeau, 2015]. Developmental neuroscience has revealed consistent patterns of structural and functional connections within the DN that correspond to the emergence and decline of core cognitive and attentional processes [Fair et al., 2008]. Moreover, aberrant DN connectivity has been linked to several psychiatric disorders, including major depression, schizophrenia, and autism [Menon, 2011].

Despite large literatures on developmental and disease‐related influences on the DN, relatively little is known about normal variation in DN functioning. Emerging evidence, however, suggests that stable personality traits predict individual differences in structural and functional properties of discrete DN regions. For example, DeYoung et al. reported associations between DN regions and the Big Five personality traits (i.e., Neuroticism, Extraversion, Openness to Experience, Agreeableness, and Conscientiousness) [Adelstein et al., 2011; DeYoung et al., 2010]. Moreover, individual differences in the frequency and phenomenology of self‐reported mind‐wandering episodes has been linked to resting‐state connectivity within core DN regions [Andrews‐Hanna et al., 2014; O'Callaghan et al., 2015]. Such work suggests that normally distributed personality traits may account for individual differences in DN functioning. However, because discrete DN regions are related to a wide range of cognitive, behavioral, and emotional variables, it remains unclear whether such traits affect global DN functioning, or whether they are uniquely related to individual DN regions.

Here, we explore whether individual differences in personality could account for variation in global DN functioning. We were particularly interested in the personality trait Openness to Experience, which is one of the so‐called Big Five personality traits that capture the major dimension of covariation among all more specific personality traits. Openness is a normally distributed personality trait reflecting the tendency to engage in imaginative, creative, and abstract cognitive processes [McCrae and Costa, 1997]. Although Openness is associated with several psychological outcomes, it is perhaps most commonly linked to flexibility of behavior and cognition [DeYoung, 2015], making it a defining feature of creative individuals (e.g., artists and scientists) [Jauk et al., 2014; Kaufman, 2013]. People high in Openness consistently show superior performance on assessments of creative cognitive ability [Beaty and Silvia, 2013; DeYoung, 2015; Jauk et al., 2013]. Understanding how Openness relates to brain network functioning may provide greater insight into the neuroscience of creativity.

Recently, DeYoung [2014, 2015] suggested that Openness may be related to DN functioning because both Openness and the DN are associated with imaginative cognition. This contention has received support from personality neuroscience showing a positive correlation between Openness and individual regions of the DN [Adelstein et al., 2011]. Recent personality models suggest that this broad personality trait is comprised of two correlated but separable subdimensions—Openness and Intellect [DeYoung et al., 2007]—which predict distinct behavioral and neural outcomes [DeYoung et al., 2005, 2009; Kaufman et al., in press]. This work thus sought to determine whether Openness and Intellect are differentially related to global functioning of the DN^1^.

To this end, we applied graph theory‐based methods to resting‐state fMRI data to assess individual differences in DN functioning. Several graph metrics have been developed to computationally assess topological properties of complex systems, including structural and functional brain networks [Medaglia et al., 2015]. One such measure is global efficiency [Latora and Marchiori, 2001]. In neurocognitive networks, this measure is thought to reflect efficiency of information processing [Achard and Bullmore, 2007]. Variation in global brain network efficiency has previously been linked to a range of individual difference variables, including intelligence, spatial orientation, memory retrieval, mathematical abilities, and creativity [Alavash et al., 2015; Arnold et al., 2013; Klados et al., 2013; Langer et al., 2012; Ryman et al., 2014; van den Heuvel et al., 2009].

We obtained personality and resting‐state fMRI data from healthy adult participants recruited from the United States and Europe. Two studies explored the role of Openness in DN global efficiency. Study 1 sought to establish the effect of Openness in DN efficiency. In Study 2, we attempted to replicate and extend this effect by accounting for individual differences in intelligence, a variable with known links to brain structure and function [van den Heuvel et al., 2009]. We hypothesized that trait levels of Openness would predict increased DN global efficiency, in light of past research linking individual DN regions to cognitive processes central to this personality trait.

## STUDY 1

### Participants

The sample consisted of 68 young adults from the University of North Carolina at Greensboro (UNCG; 41 females; mean age: 20.60, age range: 18–43). Participants received cash payment or optional course credit for their involvement in the study. All participants were right‐handed with normal or corrected‐to‐normal vision, and they reported no history of neurological disorder. The study was approved by UNCG's Institutional Review Board.

### Behavioral Assessment

Participants completed the Openness/Intellect subscale of the Big Five Aspects Scale (BFAS), a well‐validated assessment of personality [DeYoung et al., 2007]. The scale measures two aspects of the Big Five (10 items each): Openness to Experience (a trait characterized by cognitive engagement with perception, fantasy, aesthetics, and emotions) and Intellect (a trait characterized by cognitive engagement with abstract reasoning and complex problem solving) [DeYoung et al., 2005]. Openness is assessed with items such as “I seldom daydream” (reverse scored); Intellect is assessed with items such as “I like to solve complex problems.” Although these two aspects are significantly associated with each other, previous neuroimaging and behavioral research indicates that they predict distinct outcomes [DeYoung et al., 2009; Kaufman et al., in press].

### MRI Data Acquisition and Preprocessing

Resting‐state functional imaging data were acquired for five minutes as participants relaxed in the scanner with eyes closed. Whole‐brain imaging was performed on a 3T Siemens Magnetom MRI system (Siemens Medical Systems, Erlangen, Germany) using a 16‐channel head coil. BOLD‐sensitive T2*‐weighted functional images were acquired using a single shot gradient‐echo EPI pulse sequence (TR = 2000 ms, TE = 30 ms, flip angle = 78°, 32 axial slices, 3.5 × 3.5 × 4.0 mm, distance factor 0%, FoV = 192 × 192 mm, interleaved slice ordering) and corrected online for head motion. The first two volumes were discarded to allow for T1 equilibration effects.

Functional connectivity analysis was conducted using the Conn Toolbox in Matlab [Whitfield‐Gabrieli and Nieto‐Castanon, 2012]. Imaging data were slice‐time corrected using the Statistical Parametric Mapping (SPM) 8 package (Wellcome Trust Centre for Neuroimaging, London). Functional volumes were realigned, coregistered, and resliced to a voxel size of 2 mm³, normalized to the MNI template brain (Montreal Neurological Institute), and smoothed with an 8 mm^3^ isotropic Gaussian kernel. Additional preprocessing steps included low frequency filtering (0.008 – 0.09 Hz) and identification of artefactual variables via principal components analysis, including segmented white matter (WM) and cerebrospinal fluid (CSF) [Behzadi et al., 2007]. WM, CSF, and realignment parameters were regressed from the BOLD signal in a first‐level analysis.

### Network Construction

**Figure 1:**
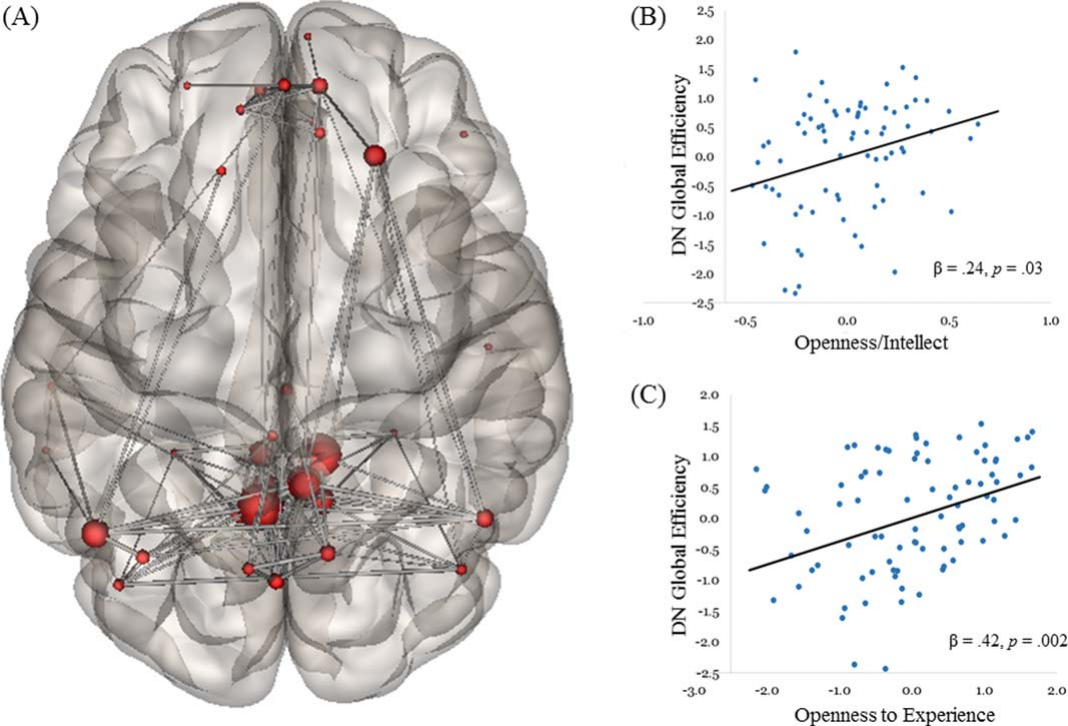
Graph analysis of DN efficiency and its relation to Openness to Experience. **A**: Group‐level depiction of nodes and edges used to define the DN. **B**: Scatterplot showing the relation between latent Openness/Intellect and DN global efficiency, controlling for age and gender. **C**: Scatterplot showing the relation between latent Openness to Experience and DN global efficiency, controlling for age, gender, intelligence, and other personality factors. Note: All variables were standardized by Z‐transformation. [Color figure can be viewed in the online issue, which is available at http://wileyonlinelibrary.com.]

The DN was defined as a graph consisting of 34 regions of interest (ROI) derived from anatomical coordinates in a meta‐analysis of resting‐state networks [see Dosenbach et al., 2010, Supporting Information material). These ROIs thus represented DN “nodes” and correlations between nodes represented network “edges” (i.e., functional connections; see Fig. 1A). We formed 5mm spherical ROIs based on the MNI coordinates from Dosenbach et al. [2010], and extracted mean time series from each ROI for graph analysis. Temporal correlations were computed between all pairs of ROIs, resulting in a 34 × 34 correlation matrix for each participant. We computed adjacency matrices from the correlation matrices, and applied a conventional network threshold (i.e., wiring cost, *K* = 0.15) to account for spurious connections, resulting in a binary undirected graph. DN integrity was assessed with global efficiency, which is mathematically expressed as the inverse of the average shortest path length in the network (i.e., the shortest number of paths needed to traverse between any pair of nodes in the network) [Latora and Marchiori, 2001]. Global efficiency has been shown to be a robust and reliable marker of network integrity [Duda et al., 2014; Medaglia et al., 2015].

### Structural Equation Modeling (SEM)

We examined the relationship between Openness/Intellect and DN efficiency using SEM. SEM employs latent variables to model error variance separately from true measurement variance [Skrondahl and Rabe‐Hesketh, 2004]. The 10 items from each facet were averaged to form two composite variables (i.e., Openness and Intellect). We formed a latent variable by specifying the two facets as indicators, which were then used to predict global efficiency of the DN. For model identification, the paths of the latent variable indicators were constrained to be equal, and the variance of each latent variable was fixed to 1 [Kline, 2011]. All analyses were conducted with Mplus 7.2 using maximum likelihood estimation with robust standard errors. Regression coefficients reported below are standardized. Because Openness and Intellect predict different outcomes [DeYoung et al., 2009; Kaufman et al., in press], we also assessed the effects of each composite variable in separate univariate analyses.

### Results

Our first model assessed the association of the latent Openness/Intellect variable with global efficiency of the DN. SEMs revealed a significant effect of latent Openness/Intellect on global efficiency (*β* = 0.25, *P* = 0.03): as Openness/Intellect increased, the DN showed more efficient information processing (see Fig. 1B). We then added age and gender to the model with Openness/Intellect predicting DN efficiency. The effect of Openness/Intellect remained stable (*β* = 0.25, *P* = 0.03); the effects of age and gender were near zero and nonsignificant (*P*'s > 0.7).

We then assessed the individual facets effects' on DN efficiency. Openness and Intellect were highly correlated (*r* = 0.61), so separate regressions were conducted to avoid collinearity in the model^2^. Univariate analyses revealed a significant effect of Intellect (*β* = 0.23, *P* = 0.04) and a nonsignificant effect of Openness (*β* = 0.16, *P* = 0.11). Consistent with the above analysis, age, and gender showed small and non‐significant effects in both models, and they did not affect the magnitude of the Openness or Intellect effects on DN efficiency.

## STUDY 2

Study 2 sought to replicate and extend the effect of Openness on DN functioning, using a different personality assessment in an independent and culturally distinct sample of participants. We also sought to determine whether other major personality variables were related to DN efficiency (i.e., Neuroticism, Extraversion, Agreeableness, and Conscientiousness), or whether the effect was specific to Openness. In addition, we sought evidence for incremental validity of Openness on the DN by assessing individual differences in intelligence—a potential “third variable” that could account for variance in DN efficiency—because intelligence has established links to brain structure and function [van den Heuvel et al., 2009].

### Participants

The sample consisted of 86 healthy adults from the University of Graz, Austria and the surrounding community (49 females; mean age: 30.35, age range: 18–49). Participants received cash payment for their involvement in the study. All participants were right‐handed with normal or corrected‐to‐normal vision, and reported no history of neurological disorder. The study was approved by the University of Graz ethics committee.

### Behavioral Assessment

Participants completed a German version of the big‐five structure inventory (BFSI) [Arendasy et al., 2011]. The BFSI assesses six facets of each of the Big Five traits (Neuroticism, Extraversion, Openness to Experience, Agreeableness, and Conscientiousness; 10 items per facet). Participants used a 1 (strongly disagree) to 5 (strongly agree) scale to indicate their level of agreement with a series of statements. The BFSI has demonstrated good reliability and validity, and it correlates highly with the German version of the NEO‐Five Factor Inventory [Arendasy et al., 2011]. Participants also completed three intelligence tests from the Intelligence Structure Battery: figural‐inductive reasoning, verbal short‐term memory, and arithmetic flexibility [see Jauk et al., 2013].

### MRI Data Acquisition and Preprocessing

Resting‐state functional imaging data were acquired for five minutes as participants relaxed in the scanner with eyes closed. Whole‐brain imaging was performed on a 3T Siemens Skyra MRI system (Siemens Medical Systems, Erlangen, Germany) using a 32‐channel head coil. BOLD‐sensitive T2*‐weighted functional images were acquired using a single shot gradient‐echo EPI pulse sequence (TR = 2500 ms, TE = 27 ms, flip angle = 90°, 32 axial slices, 4.0 × 4.0 × 4.0 mm, distance factor 25%, FoV = 256 × 256 mm, interleaved slice ordering) and corrected online for head motion. The first two volumes were discarded to allow for T1 equilibration effects. Head motion was restricted using firm padding surrounding the head. Following functional imaging, a high resolution T1 scan was acquired for anatomic normalization. Preprocessing of functional and anatomical data followed the same procedure outlined in Study 1.

### Structural Equation Modeling

**Figure 2:**
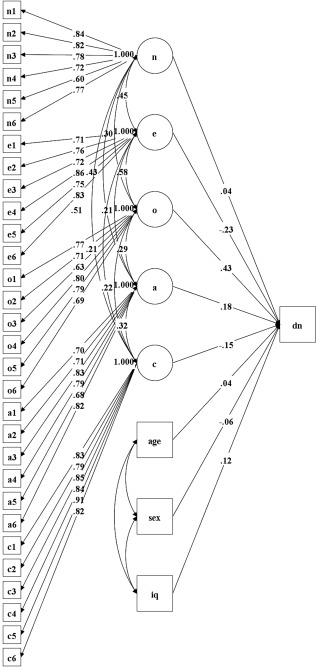
SEM from Study 2 showing effects of the latent personality variables on DN efficiency. All paths are standardized coefficients. A: Agreeableness; C: Conscientiousness; DN: Default Network; E: Extraversion; N: Neuroticism (emotional stability); O: Openness to Experience.

The six facets of each personality variable were specified as indicators, resulting in five latent variables for analysis (i.e., Neuroticism, Extraversion, Openness, Agreeableness, and Conscientiousness; see Fig. 2). The three intelligence test were averaged to form a composite variable. Intelligence was not modeled as a latent variable because the total number of latent variable indicators would have exceeded the number of estimated model parameters, leading to model convergence issues [Kline, 2011]. Intelligence, age, and gender were modeled as observed variables.

## RESULTS

### Personality and the DN

We first examined the associations of the Big Five traits with global efficiency of the DN. Results revealed a large effect of Openness to Experience on global efficiency (*β* = 0.43, *P* = 0.001; see Fig. 1C). Openness was the only significant predictor of DN efficiency, although Extraversion, Agreeableness, and Conscientiousness showed small and marginal effects. We then added age, gender, and intelligence to the model with personality predicting DN global efficiency. The effect of Openness remained robust (*β* = 0.43, *P* = 0.001); age, gender, and intelligence showed small and nonsignificant effects on DN global efficiency.

To explore facet‐level effects, we ran separate regression models with each of the six Openness facets (Openness to Fantasy, Aesthetics, Feelings, Actions, Ideas, and Values) predicting DN global efficiency, controlling for age, gender, and intelligence. All facets showed significant effects on DN efficiency, with the exception of Aesthetics (*β* = 0.14, *P* = 0.13)—Fantasy (*β* = 0.32, *P* = 0.005), Feelings (*β* = 0.24 *P* = 0.02), Actions (*β* = 0.26, *P* = 0.02), Ideas (*β* = 0.25, *P* = 0.01), and Values (*β* = 0.31, *P* = 0.001).

## DISCUSSION

Despite much research on the development and disorder of the DN, little is known about traits associated with normal variation in this core functional network. Here, we show for the first time that the functional organization of the DN is related to individual differences in Openness to Experience—a normally distributed personality trait associated with imaginative, creative, and intellectual cognitive processes. Study 1 established the effect of Openness on DN global efficiency; Study 2 replicated and extended this effect using a separate personality assessment in a culturally distinct population, controlling for several factors related to personality and brain function (i.e., intelligence, age, and gender). Taken together, these findings point to a biological basis of Openness to Experience, and suggest that normally distributed personality variables affect the functional organization of large‐scale brain networks.

This work extends previous studies showing associations among personality factors and individual DN regions. We used graph theory methods to model a whole‐brain network of DN regions, thus permitting a fine‐grained look at whether global network functioning is sensitive to variation in Openness to Experience, a core personality trait reflected in the general population. Our findings build on recent work using graph theory modeling of whole‐brain structural and functional networks examining contributions of cognitive and personality factors [Davis et al., 2013; Sampaio et al., 2014; Servaas et al., 2014; van den Heuvel et al., 2009]. For example, van den Heuvel et al. [2009] found that fluid intelligence was inversely related to characteristic path length within a whole‐brain functional network, pointing to an important role of individual differences in cognitive ability in predicting brain network efficiency. Such findings provide compelling evidence for a role of cognitive, personality, and creativity variables in shaping structural and functional brain systems [Beaty et al., 2014; Faust and Kenett, 2014; Kenett et al., 2014; Medaglia et al., 2015; Ryman et al., 2014; van den Heuvel et al., 2009]. Future research should continue to explore factors underlying variation in neurocognitive network topology.

This study suggests that high‐Openness is related to a more efficiently functioning DN. We suspect that this relationship is due in part to the imaginative characteristics that define both Openness and the DN. Our findings are consistent with a large literature on the role of the DN in self‐generated thought, such as mind‐wandering, future thinking, and creative idea production [Andrews‐Hanna et al., 2014; Beaty et al., in press]. Thus, a more efficiently functioning DN may allow people high in Openness to more effectively engage the neurocognitive processes associated with this network. A defining feature of Openness is creativity—not only are Open people more likely to self‐report high levels of imagination [DeYoung, 2014, 2015], they also show superior performance on cognitive tasks that measure creative thinking [Beaty and Silvia, 2013; Jauk et al., 2014]. In this context, the ability to efficiently access the neurocognitive resources of the DN may partially account for the ability of highly Open people to generate creative ideas.

Past research suggests that Openness is prone to change across development. Most notably, developmental research has reported a relative decline in self‐reported Openness in older adults [Roberts et al., 2006]. An interesting question for future research is to determine whether this age‐related decline in Openness corresponds to decreased DN efficiency. Another open question is whether Openness underlies core mechanisms involved in domain‐general imaginative processes, or whether its role is more specific to select features of the DN (e.g., future thinking, mental simulation, and creative cognition). This question could be addressed with task‐based fMRI by assessing DN efficiency during various tasks requiring imaginative and sensory processing in a high‐Openness sample [cf. Passamonti et al., 2015], or by exploring whether Openness and related cognitive abilities modulate brain network connectivity during cognitive tasks [Beaty et al., 2015]. Such approaches can shed light on the extent to which normally distributed cognitive and personality traits affect the intrinsic architecture of large‐scale brain systems.

## Footnotes

1 We use the label “Openness” to refer to the broader Big Five dimension encompassing both Openness and Intellect, except for when we discuss the distinct Openness subscale in Study 1.

2 A hierarchical regression analysis was also conducted to determine whether Openness and Intellect differentially predict default network efficiency. Age and gender were entered in a first block, and the two scales were entered in a second block. Results showed nonsignificant effects of both Openness and Intellect on default network efficiency; moreover, the change in R‐squared was not significant.
